# Study on the Effect of Nanoparticle Used in Nano-Fluid Flooding on Droplet–Interface Electro-Coalescence

**DOI:** 10.3390/nano11071764

**Published:** 2021-07-06

**Authors:** Donghai Yang, Huayao Sun, Qing Chang, Yongxiang Sun, Limin He

**Affiliations:** 1 College of Pipeline and Civil Engineering, China University of Petroleum, Qingdao 266580, China; S20060058@s.upc.edu.cn (H.S.); y.x.sun@hotmail.com (Y.S.); helimin@upc.edu.cn (L.H.); 2 Surface Engineering Pilot Test Center, CNPC, Daqing 163000, China; 3 China Petroleum Pipeline Engineering Corporation Shanghai Branch, Shanghai 200127, China; cppechangqing@cnpc.com.cn

**Keywords:** electro-coalescence, nanoparticle, droplet, interfacial tension, conductivity

## Abstract

Nano-fluid flooding is a new method capable of improving oil recovery; however, nanoparticles (NPs) significantly affect electric dehydration, which has rarely been investigated. The effect of silica (SiO_2_) NPs on the droplet–interface coalescence was investigated using a high-speed digital camera under an electric field. The droplet experienced a fall, coalescence, and secondary droplet formation. The results revealed that the oil–water interfacial tension and water conductivity changed because of the SiO_2_ NPs. The decrease of interfacial tension facilitated droplet deformation during the falling process. However, with the increase of particle concentration, the formed particle film inhibited the droplet deformation degree. Droplet and interface are connected by a liquid bridge during coalescence, and the NP concentration also resulted in the shape of this liquid bridge changing. The increase of NP concentration inhibited the horizontal contraction of the liquid bridge while promoting vertical collapse. As a result, it did not facilitate secondary droplet formation. Moreover, the droplet falling velocity decreased, while the rising velocity of the secondary droplet increased. Additionally, the inverse calculation of the force balance equation showed that the charge of the secondary droplet also increased. This is attributed to nanoparticle accumulation, which resulted in charge accumulation on the top of the droplet.

## 1. Introduction

With the rapid development of nanotechnology, nanoparticles (NPs) have been widely applied in industries including food, biomedicine, textiles, electronics, machinery, chemistry, petroleum, etc. [1,2]. In particular, their applications have pierced through different petroleum disciplines from exploration to drilling, exploitation, and reprocessing [3]. In recent years, nano-fluids have been proven to enhance oil recovery. In terms of the economy, silicon dioxide (SiO_2_) NPs are the most commonly used NPs in many industrial products. Compared to conventional flooding methods such as polymer flooding, chemical flooding, thermal flooding, surfactant flooding, and so on [4], nano-fluid flooding has more advantages in terms of a strong breakthrough ability with little damage to the structure, stable fluctuation of differential pressure, and high oil recovery [5].

Injected SiO_2_ nano-fluids can be re-extracted from the ground along with oil to form oil–water mixtures containing NPs during crude oil extraction. The shear forces of transporting such mixtures in pipelines significantly promotes the formation of oil–water emulsions, and the presence of other substances such as fine particles and salts may further improve the stability of emulsions [6,7,8,9,10].

To meet the requirement for the subsequent oil refining process, effective dehydration is needed to remove water from oil. It is well known that NPs can stabilize oil–water emulsions by changing the surface tension, conductivity, and fluid viscosity during the extraction and transportation processes [11,12,13]. Nonetheless, owing to the extremely high surface energy, NPs are commonly highly charged, which results in elevated liquid conductivity and the short circuiting of electrodes. As a result, NPs pose challenges for current dewatering technologies including electrostatic demulsification, filtration, pH adjustment, heat treatment, and the addition of chemical demulsifiers [14,15,16,17]. Among the previously mentioned technologies, electrostatic demulsification is the technology of particular concern because of its reliability, energy efficiency, and effectiveness [15,18,19].

However, the existence of NPs would make the electrostatic demulsification, especially coalescence process of droplets, more complex.

### 1.1. Droplet–Interface Coalescence

In an electric dehydrator, two main coalescence processes commonly occur: the coalescence between the droplets in the emulsion and the droplet–interface coalescence between the droplets and the water layer that has settled at the bottom. For the latter, a planar interface with a certain thickness is formed by the gradual settling down of the large droplets through the water-in-oil emulsions followed by connection with the water layer. Drop-interface coalescence then exists under an electric field.

During the droplet–interface electro-coalescence, a droplet rests on the interface and is separated with a thin oil film. The droplet and interface are then connected by a ‘liquid bridge’ formed due to a strong attractive electrical force between the bottom of the droplet and the interface [20]. As a channel, this ‘liquid bridge’ plays an important role in the charge and water transfer. When the oil film ruptures, the droplet acquires the same potential as the interface because of the redistribution of charges through the ‘bridge’. After contact, the downward electrostatic attraction vanishes while upward electric force applied by an external electric field continues stretching the droplet to hinder the droplet–interface coalescence process [21]. The force acting on the droplet also includes the buoyancy force *F*_B_, drag force *F*_D_, gravity force *G*, and additional pressure [22,23]. All of these forces together determine the pattern of droplet–interface coalescence. Under these forces, first, the ‘liquid bridge’ expands rapidly and then contracts when it nearly reaches the same width as the water droplet. Moreover, the height of droplet also gradually decreases [24]. The following two coalescence patterns exist: complete coalescence, i.e., the droplet completely merges with the bulk water and no smaller droplets are left over the interface and partial coalescence, wherein small secondary droplets are left behind [24,25,26,27,28]. Additionally, the necking time that finally sets the secondary droplet size is shown to be principally governed by the outer phase viscosity to interfacial tension ratio [29]. Obviously, partial coalescence is undesirable for electrostatic dehydration because it is more difficult to remove the smaller droplets.

### 1.2. Influence of NPs on Droplet–Interface Coalescence

The presence of NPs has an influence on the droplet coalescence. For example, Binks et al. [30] investigated water in oil emulsions that were stabilized with hydroplethobic SiO_2_ NPs and proposed that the formation of a film structure of NPs at the oil–water interface resulted in the dispersed phase droplets being very stable in the emulsion. Additionally, Vignati et al. [31] found that droplets with low interfacial particle coverage were more stable in the emulsion. They attributed this result to the redistribution of particles on the surface of the droplets when the droplets approached each other. However, Bremond et al. [32] found that the separation of emulsion droplets favored coalescence because of the formation of two nipples that were facing each other in the contact area that hastened the connection of the interfaces.

Under an electric field, the NPs may move and redistribute themselves around the interface, thereby changing the interface characteristics of the droplets. Liu et al. [33] found that two contacting emulsion droplets can be forced to coalesce into a combined one when the applied voltage is above a critical value. Flatte et al. [34] and Musinski et al. [35] confirmed that the electric field could drive the NPs to the oil–water interface. Cui et al. [36] found that the droplet deformed when the electric field was applied, which increased the surface area and resulted in the adsorption of more NPs on the droplet surface. Chen et al. [37] found that with the increase of the electric field intensity, cone-shaped protrusions formed at the defect position of the interfacial particle film, forming a liquid bridge and finally coalescing. However, due to the presence of particles, the reduction in surface area may cause particle clogging, and thus result in the stagnation of coalescence. Pawar et al. [38] observed the phenomenon of coalescence stagnation in their experiment. They found that for the coalescence between two identical droplets, when the total particle interface coverage of the droplets was between 1.43 and 1.81, stagnation occurred. For other total particle interface coverages, either complete coalescence or non-coalescence occurred.

Recently, extensive research efforts have been devoted to the study of the effect of NPs on droplet–interface coalescence. For example, Harbottle et al. [39] studied the coalescence between droplets containing SiO_2_ NPs and an interface in the absence of an electric field. The results showed that the increase of particle concentration and interaction energy between particles could change the coalescence mode from partial coalescence to complete coalescence. Furthermore, Jaeger et al. [40] simulated the effects of particle wettability and volume fraction on the growth and fracture of liquid bridges during droplet–interface coalescence. The results indicated the possibility of the accumulation of particles on the surface and internal area of the liquid bridge during coalescence, which affected its fracture. Yang et al. [41] studied the coalescence of nanoparticle-laden droplets resting on the oil–water interface under an electric field and found that increasing the particle concentration could decrease the size of the secondary droplets formed during partial coalescence.

In a word, the current study of NPs on droplet–interface coalescence focuses on patterns under different conditions and rarely on the topological changes of the liquid bridge and charges of secondary droplets. In this study, the deformation, migration, and coalescence of droplets with different NP concentrations were studied under electric fields. Moreover, the study focuses on the topological changes of the liquid bridge in the horizontal and vertical directions as well as the rising velocity and the charge of secondary droplets. The underlying mechanism of NP on the droplet–interface coalescence was analyzed, including the competitive effects between interfacial tension and interfacial film as well as conductivity. The results of this study may be useful for the development of electric dehydration facilities.

## 2. Experimental Set-Up and Procedure

### 2.1. Experimental Cell

**Figure 1:**
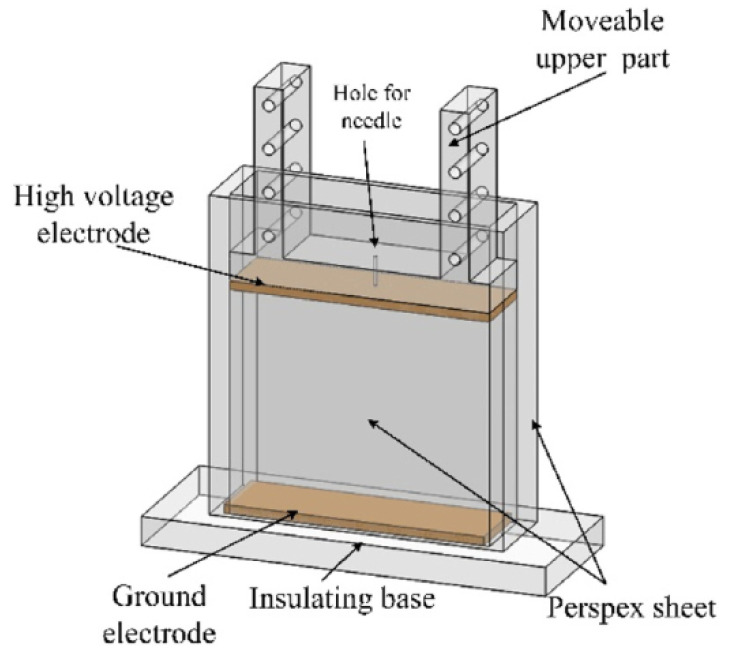
Schematic diagram of experimental cell.

The structure of the experimental cell, which is similar to that used before [42], is shown in Figure 1. To facilitate the observation of the experimental process, the cell was made of transparent Perspex. Brass plates with the dimensions of 90 mm × 25 mm were polished, as were the electrodes, which were arranged parallel. A high voltage electrode was attached to the bottom of the slider, which could change the distance (in this study, the distance is 64 mm) between the two electrodes. Furthermore, a ground electrode was fixed at the insulating base. A small hole with a diameter of 1 mm through the mid-point of the movable slider enabled a hypodermic needle to pass through. The needle was attached to a syringe (Hamilton micro-liter syringe) and used to introduce small aqueous droplets into the cell, the radii of which ranged from 914 to 1240 µm. The released position of the droplets was the same. When the bottom of the droplet reached a specific position, the power was turned on to apply the electric field. The electric field aided in the acceleration of the migration and coalescence of the droplet.

An electric field was applied between the high voltage electrode and the ground electrode by connecting to a high voltage power amplifier (TREK 20/20C, New York, NY, USA). A high-speed camera (NAC Hotshot 1280, nac Americas Inc., Salem, MA, USA) utilizing a frame rate of 1000 fps equipped with a 100 × lens (Mitutoyo 5 × objective, Kawasaki, Japan; with a 20× tube made by Pomeas, Dongguan, China) was used to observe and record the coalescence process between the droplet and the interface. A halogen lamp with four flexible fiber optic heads was used for lighting and to facilitate in the focusing of the lighting intensity for accurate adjustments. The experiments were performed at 22 ± 1 °C.

### 2.2. Preparation of Experimental Liquids

In our experiments, the bottom half of the cell was filled with nano-fluids and the top half was filled with dimethyl silicone oil to form two-phase interface. The droplet injected into the cell was the same liquid as the lower medium.

**Figure 2:**
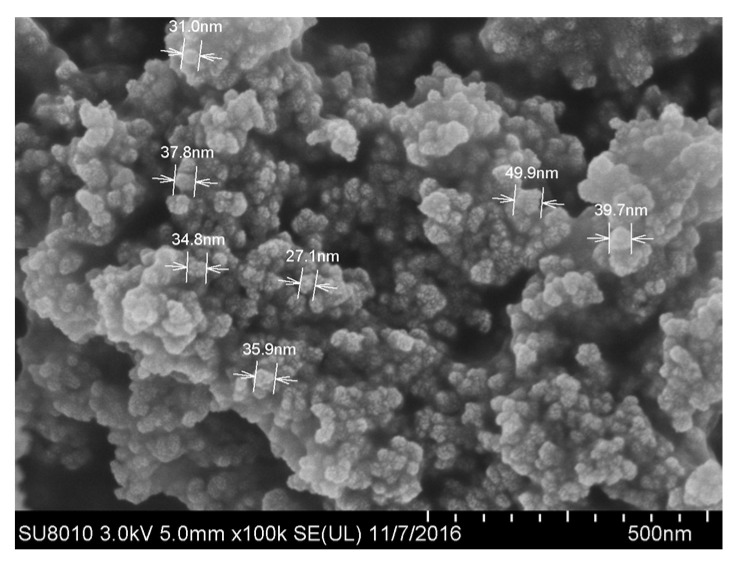
Morphology of NPs [43].

**Figure 3:**
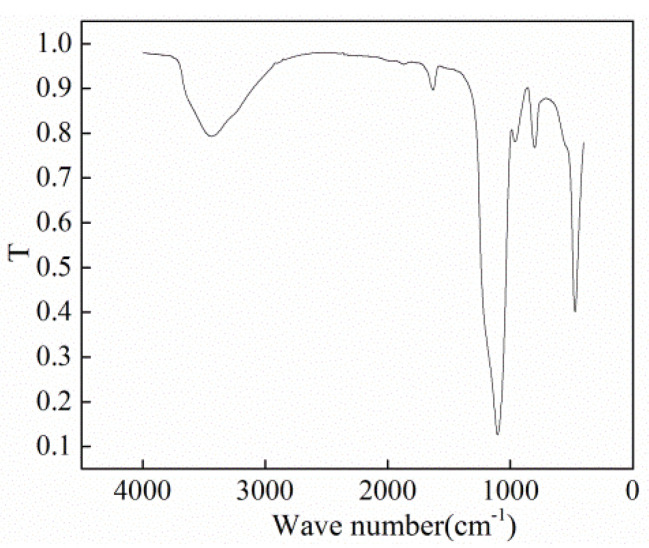
Infrared spectrogram of NPs [43].

To prepare nano-fluids, SiO_2_ NPs and deionized water were used. The size of the SiO_2_ NPs was measured using scanning electron microscopy (SEM, Hitachi SU8010, Tokyo, Japan), and the size was found to be about 30 nm (±10 nm). X-ray diffraction (XRD, Tensor II, Bruker Optics, Ettlingen, Germany) was employed to detect the chemical groups on the surface of the NPs. The results are shown in Figure 2 and Figure 3, respectively. Through comparison with the Standard Infrared Spectrum Atlas, the characterization results confirmed that the wide absorption peak at 3439.88 cm^−1^ corresponded to the antisymmetric and symmetrical stretching vibrations of the –OH group. The strong absorption band at 1100 cm^−1^ is attributed to Si–O–Si antisymmetric stretching vibration. The peak at 958 cm^−1^ belongs to the bending vibration absorption peak of Si–OH, and the peaks at 800 and 469 cm^−1^ correspond to the symmetrical stretching and bending vibrations of Si–O bond. The deionized water was produced using Millipore ultrapure water systems. In experiments, the mass concentration of SiO_2_ NPs was 0.5%, 1%, and 2%, respectively.

For the effective and sufficient dispersion of the NPs into the water, ultrasonic vibration (ultrasonic processor FS-1200 N, Shang Hai Sheng-xi Co. Ltd., Shanghai, China) was used for about 20 min. Further, a magnetic stirring apparatus (IKA Maxi MR 1, Staufen, Germany) was used to continuously stir the nano-fluids to prevent the reunion of the NPs.

The conductivity of the aqueous phase and the oil phase was measured using a Rex conductivity meter and an oil conductivity meter (GD29YX1154B, Beijing, China), respectively. The viscosity of the liquids was measured using an Anton Paar Physica MCR102 (Graz, Austria) Moreover, the interfacial tension was measured using a fully automatic surface and interface tensiometer (USA KINO Industry, Boston, MD, USA). Density was measured using a volumetric flask. The physical properties of the oil, deionized water, and SiO_2_ nano-fluids are listed in Table 1. It should be noted that the 0.5, 1, and 2 wt% in the table and the wt% in the following text refer to the mass concentration of SiO_2_ NPs.

### 2.3. Data Extraction and Processing

**Figure 4:**
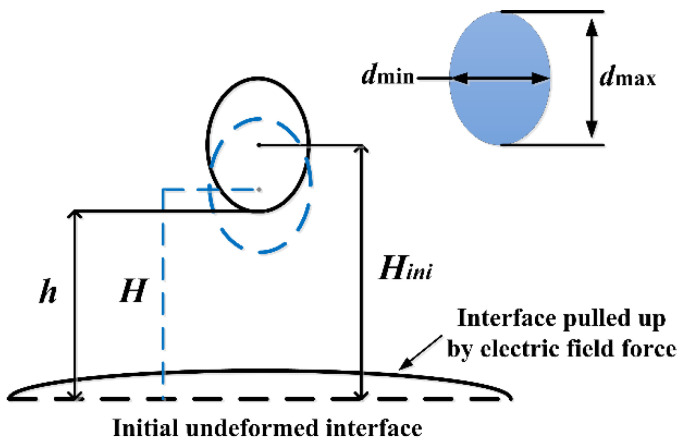
Schematic diagram of parameters extracted from the falling droplet.

Figure 4 shows the characteristics of deformation and migration, which were studied by defining the aspect ratio of the droplet *d*_max_/*d*_min_, which quantitatively represents the deformation degree of the falling droplet.

Between them, *d*max and *d*min represent the length of the long axis and the short axis of the droplet, respectively. *H*/*H*_ini_ is defined as the dimensionless height of the droplet center, where H and *H*_ini_ are the real-time distance and the initial distance between the droplet center and the undeformed oil–water interface, respectively, and quantitatively reflect the vertical displacement of the falling droplet. Moreover, *h* represents the distance between the bottom of the falling droplet and the initial undeformed oil–water interface at the moment the electric field was applied.

**Figure 5:**
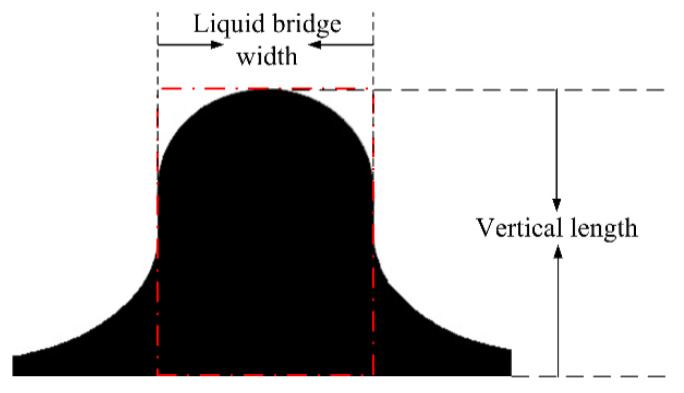
Schematic illustration of typical parameters of the droplet shape.

For droplet–interface coalescence, owing to the asymmetry between the upper and lower agglomerates and the dynamic change in the morphological characteristics of the coalescence droplet, the development of the droplet morphology was difficult to describe. Figure 5 shows the typical morphology of coalesced droplets, where the red dotted line in the figure indicates the liquid bridge area. In this study, the characteristic parameters that define the shape of the droplet include the liquid bridge width, which corresponds to the length of the rectangular structure shown in red dotted line, which is used to describe the expansion and contraction of the liquid bridge in the horizontal direction. The droplet apex height represents the distance from the highest point of droplet to the undeformed oil–water surface, which is used to reflect the change in droplet height in the vertical direction. For more convenient comparative analysis, the instantaneous liquid bridge width and the droplet apex height were kept dimensionless using the diameter and the initial droplet apex height, respectively, which are defined as the dimensionless liquid bridge width (*W*_liquid bridge width_/*D*) and the dimensionless droplet apex height (*H*_vertical length_/*H*_initial vertical length_), respectively.

## 3. Theory

Owing to the difference in permittivity, the water droplets in the oil become polarized through the applied electric field to form a dipole. Therefore, the droplets are stretched by the electric field force and become deformed, and the horizontal oil–water interface is also raised slightly upwards by the pulling force of the applied electric field. At the same time, under the action of gravitational force and the electrostatic attraction between the droplet and the interface, the droplet gradually moves to the oil–water interface. Eventually, the dynamic behavior of the falling droplet affects droplet–interface coalescence.

Assuming that the positive direction is vertically downward, the expression for the movement of the falling droplet can be derived from Newton’s second law as follows:

(1) $$\frac{dx}{dt}=v$$

(2) $$\frac{dv}{dt}{=F}_{r}+G-F_{b}-F_{d}-F_{f}-F_{\mathrm{vm}}-F_{E}$$

where *F*_f_ is the film-thinning force, *F*_r_ denotes the droplet–interface radial electrostatic attraction, and both belong to the droplet–interface force. When the droplet falls to the oil–water interface, the oil film between the droplet and the interface continuously drains and becomes thin, and the film-thinning force (*F*_f_) of the liquid film hinders the drainage of the oil film. Furthermore, when the oil film between the droplet and the interface is thinned to a certain extent, *F*_f_ of the liquid film becomes significant. For the proximity between the two droplets, the reduced radius *a* = 2*r*_1_*r*_2_/(*r*_1_ + *r*_2_) is defined. Assuming that the droplet is rigid, *F*_f_ can be expressed as follows [44]:

(3) $$F_{f}=\frac{6\pi \mu_{c}a^{2}v}{h}$$

In this study, the oil–water interface can be regarded as the surface of a droplet whose radius *r*_2_ tends to be infinite. *r*_2_ is much larger than *r*_1_; therefore, the process of droplets falling onto the interface can be simplified as the coalescence of pairs of unequal droplets, and the converted radius *a* is approximately equal to 2*r*_1_. Therefore, for the droplet–interface coalescence system, Equation (3) can be simplified as follows:

(4) $$F_{f}=\frac{24\pi \mu_{c}r_{1}{}^{2}v}{h}$$

When the distance between the centers of the two droplets is too far (*d* >> *a*), electrostatic attraction *F*_r_ conforms to the point-dipole model. In contrast when the droplet pairs are close to each other (*d* < *a*), the influence of the droplet size cannot be ignored. At this time, the electrostatic attraction *F*_r_ conforms to the dipole-induced dipole model.

Specifically, the expression of the point-dipole model is as follows [45]:

(5) $$F_{r}=12\pi \beta^{2}\varepsilon_{\mathrm{oil}}\left|E^{2}\right|r_{1}{}^{3}r_{2}{}^{3}{\left|d\right|}^{-4}\left({3\cos}^{2}\theta -1\right)$$

where, *θ* is the angle between the center line of the droplets and the direction of the applied electric field. In this study, *θ* = 0, and *d* represents the center distance of the droplets.

The expression of the dipole–induced-dipole model [46] is presented as follows:

(6) $$F_{r}=12\pi \beta^{2}\varepsilon_{\mathrm{oil}}\left|E^{2}\right|r_{1}{}^{3}r_{2}{}^{3}{\left|d\right|}^{-4}\left({3K}_{1}\cos^{2}\theta -1\right)$$

(7) $$K_{1}=1+\frac{{\beta r}_{1}{}^{3}{\left|d\right|}^{5}}{{\left({\left|d\right|}^{2}-r_{2}{}^{2}\right)}^{4}}+\frac{{\beta r}_{2}{}^{3}{\left|d\right|}^{5}}{{\left({\left|d\right|}^{2}-r_{1}{}^{2}\right)}^{4}}+\frac{{3\beta r}_{1}{}^{3}r_{2}{}^{3}\left(3{\left|d\right|}^{2}-{\;r}_{1}{}^{2}-{\;r}_{2}{}^{2}\right)}{{\left({\left|d\right|}^{2}-{\;r}_{1}{}^{2}-{\;r}_{2}{}^{2}\right)}^{4}}$$

When the dispersed droplets move relative to each other in the continuous phase, the droplets experience a drag force (*F*_d_) in the direction opposite to the movement, which hinders their relative movement. The expression of drag force *(F*_d_) is represented as follows:

(8) $$F_{d}=\frac{1}{2}\rho_{c}C_{d}Av^{2}$$

where, *ρ*_c_ refers to the continuous phase density; *A* refers to the projected area of the droplet; *v* refers to the relative velocity of the droplet; and *C*_d_ refers to the drag coefficient. Taking into account the influence of the droplet surface tension gradient, the expression of the drag coefficient *C*_d_ is revised to:

(9) Cd=24Re·3λ+2+2/3γ1(μcv)−13λ+3

where ${\lambda =\mu }_{d}{/\mu }_{c}$ is the ratio of the droplet viscosity *µ*_d_ to the continuous phase viscosity *µ*_c_. *γ*_1_ and Re are the interfacial tension gradient and the droplet’s Reynolds number, respectively.

The direction of the buoyancy of the droplet in the continuous phase is opposite to the direction of gravity, and the magnitude is expressed as follows:

(10) $$F_{b}{=\rho }_{c}gV_{d}$$

where g represents the acceleration due to gravity, and *V*_d_ represents the droplet volume.

The virtual mass force *F*_vm_ [47] is an unsteady force used to explain continuous phase acceleration caused by the movement of the droplets when a relative acceleration exists between the dispersed phase droplets and the continuous phase. Its expression is

(11) $$F_{\mathrm{vm}}=\frac{2}{3}{\pi r}_{1}{}^{3}\rho_{c}\left(\frac{du}{dt}-\frac{dv}{dt}\right)$$

where *r*_1_ is the droplet radius. $\frac{du}{dt}$ represents the acceleration of the continuous phase; this value is always 0 in this study. $\frac{dv}{dt}$ denotes droplet acceleration where “-” means that the direction of the force is opposite to the direction of droplet movement.

The droplets containing NPs are negatively charged because of the negative NPs. Therefore, the droplets are affected by the net electric field force, actuating the droplets to migrate. As a result, the net electric field force *F*_E_ is expected to be related to the charge amount of the droplet, and can be simply expressed as follows:

*F*_E_ = *QE* (12)

where *Q* is the charge of the droplet, and *E* is the intensity of the applied electric field.

Moreover, the droplet is affected by gravity, and the expression is:

(13) $${G=\rho }_{d}{gV}_{d}$$

## 4. Results and Discussion

**Figure 6:**
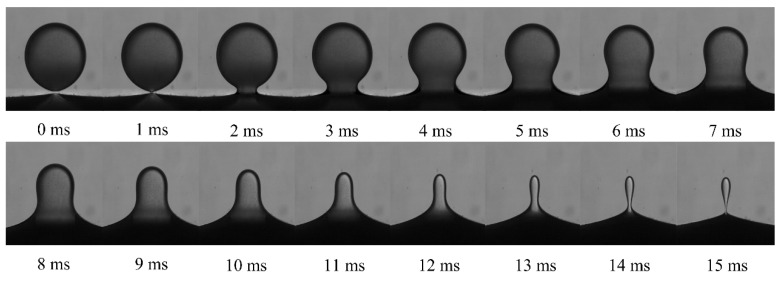
Morphological evolution during droplet–interface electro-coalescence with a particle concentration of 1 wt%.

The typical droplet–interface coalescence process observed in experiments is shown in Figure 6. In this study, the electric field strength was 256 kV m^−1^, and the droplet diameter was 1241 µm. In order to exclude the effect of height, the initial height between the droplet and the interface was fixed at 0.5 mm. As mentioned above, the NP concentration ranged from 0 to 2 wt% to study the effect of the NPs. For example, as shown in Supplementary Materials Video S1, where the NP concentration is 0.5 wt%. It showed the dynamic process of drop–interface partial coalescence, which was more visualized.

Driven by the electric field, as the falling droplets approached the oil–water interface, the oil film between the droplet and the interface was drained to rupture. The droplets and the interface made contact with each other, and a liquid bridge was formed to facilitate mass transfer and initiate coalescence.

Notably, the liquid bridge first expanded and then contracted. With mass transfer, the height of the droplet apex continued to decrease. This experimental observation indicated that when the liquid bridge was contracted to be pinched off, there was still a part of the liquid above the interface, which shrank into a spherical shape again, forming secondary droplets and partially coalescing.

**Figure 7:**
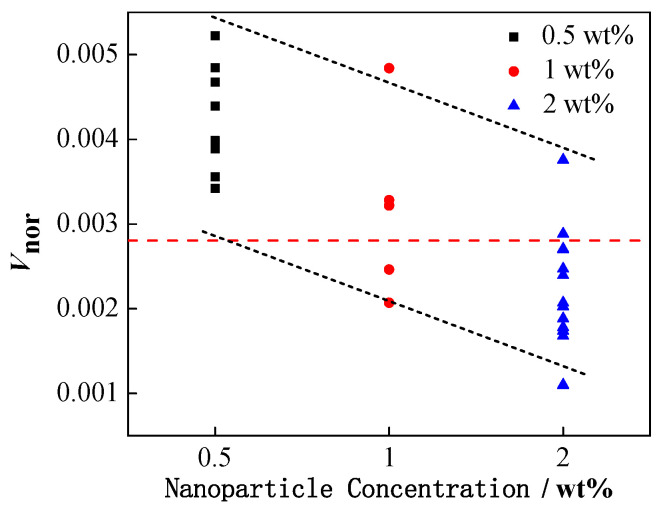
The relationship between the volume ratio of secondary and initial droplets and the concentration of NPs.

Figure 7 demonstrates the effect of NP concentration on the formation of secondary droplets during partial coalescence.

In order to normalize the volume of the formed secondary droplets in partial coalescence, the volume ratio of the secondary droplets to the initial droplet (*V*_nor_) is defined as follows:

*V*_nor_ = *V*_secondary_/*V*_initial_ (14)

The NP concentration is represented in three different color legends, and the red dotted line represents the *V*_nor_ of pure water droplets during partial coalescence. Clearly, when the NPs were added, the increased concentration of NPs could generally inhibit the formation of secondary droplets, leading to the decrease in the value of *V*_nor_.

The underlying mechanism for this result is complex. On the one hand, it is believed to be related to the adsorption film of the NPs on the oil–water interface. On the other hand, the change in the physical properties of the nanoparticle-laden droplets also significantly impacts the coalescence process. Specifically, the introduction of NPs in the water phase can simultaneously affect both the oil–water interfacial tension and conductivity of the water phase, thereby synergistically and competitively affecting coalescence. Therefore, it is necessary to comprehensively discuss the influence of each physical property.

### 4.1. Effect of Interfacial Tension

After the particles are adsorbed onto the oil–water interface, variation in the interfacial properties is observed. Similar to surfactants, NPs can adsorb onto the oil–water interface, resulting in a decrease in the interfacial tension. However, unlike surfactants, the interfacial tension does not decrease monotonically to a certain minimum with the increase of NP concentration; nonetheless, this presents a more complicated change. The following three types of changes are commonly encountered: as the concentration of NPs increases, the surface/interfacial tension decreases monotonously [48], increases monotonously [49,50], or decreases to a minimum and then increases [31,43,51,52]. In our experiments, comparative analysis of the interfacial tension for three different concentrations provides the following order of magnitude: σ_2 wt%_ > σ_1 wt%_> σ_0_._5 wt%_. The reason for this is generally believed to be related to the adsorption state of NPs on the surface/interface. In the case of a low concentration, the distribution of the NPs at the interface is relatively loose, and this may play a role in reducing the interfacial tension. Nonetheless, with the increase in the concentration, the attractive force between the particles causes the particles to pack closely together at the surface/interface, resulting in more surface/interface tension. Moreover, NPs can form a single-layer or multi-layer particle film after getting adsorbed on the interface when the concentration is further increased [53]. Compared to a pure liquid film, it has stronger mechanical strength [54] and viscoelasticity when the oil–water interface is covered with a particle film, which can hinder the deformation and collision coalescence of droplets [55,56].

**Figure 8:**
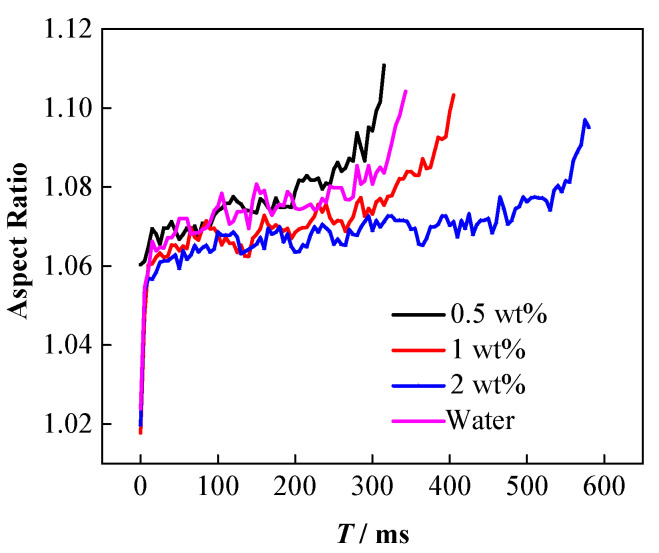
Effect of particle concentration on degree of deformation.

Theoretically, the decrease of oil–water interfacial tension leads to more significant extension of the droplets under the action of electric fields, which can be proven by the droplet deformation and droplet morphology changes that occur during coalescence. Figure 8 shows the deformation degree of the nanoparticle-laden droplets during the falling process. Similar to pure water droplets, the deformation degree of the nanoparticle-laden droplets also continues to increase dynamically during the falling process. Comparative analysis of the deformation degree of the droplet at the same time as the falling process indicates that the deformation degree of the 0.5 wt% nanoparticle-laden droplets is the largest, higher than that of pure water, followed by the 1 wt% nanoparticle-laden droplets, while the deformation degree of the 2 wt% nanoparticle-laden droplets is the smallest.

These experimental results are attributed to the adsorption of the NPs on the droplet surface. Table 1 summarizes that the adsorption of the NPs on the droplet surface can lead to irregular changes in the interfacial tension of oil and water. Specifically, the interfacial tension of 0.5 wt% nanoparticle-laden droplets in oil is the lowest, while that of the 1 wt% nanoparticle-laden droplets is higher. The interfacial tension of the 2 wt% nanoparticle-laden droplets is the highest and is close to that of pure water. The lower the interfacial tension, the easier it is for the droplet to deform [57,58,59,60]. Therefore, the 0.5 wt% nanoparticle-laden droplets are the most prone to deformation under the electric field. For the1 and 2 wt% nanoparticle-laden droplets, although the interfacial tension is lower than that of pure water droplets, the particles get adsorbed on the surface and form a rigid particle film [9], which can hinder the deformation of the droplets under electric field force, resulting in a lesser degree of deformation. Moreover, when the particle concentration increases, more particles are adsorbed on the droplet surface, and the particle film becomes more rigid; thus, the droplets are not easily deformed.

In order to verify the effect of the NPs on coalescence in different waveforms, the deformation and oscillation of the nanoparticle-laden droplets in the AC electric field were also studied.

**Figure 9:**
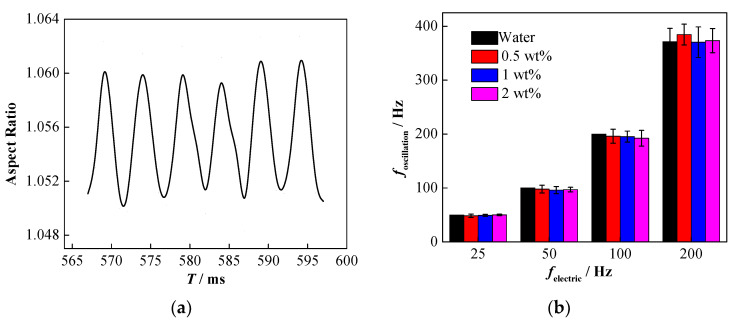
Influence of electric field frequency on the oscillatory deformation of nanoparticle-laden droplets (The RMS of electric field intensity is 192 kV·m^−1^, and the frequency is 100 Hz). (**a**) Deformation of nanoparticle-laden droplets; (**b**) effect of electric field frequency on droplet oscillation frequency.

Considering a sinusoidal AC electric field as an example, as shown in Figure 9a, the deformation of the nanoparticle-laden droplets during the falling process still accords with the sinusoidal form. The 10 *T* method (where *T* stands for the period) was used to analyze the influence of NP concentration on the droplet oscillation frequency. Specifically, the typical 10 consecutive oscillations were extracted from the data of each set of experiments, the total time (i.e., 10 *T*) required by the droplet to oscillate once was then recorded, and the droplet oscillation frequency was determined.

**Figure 10:**
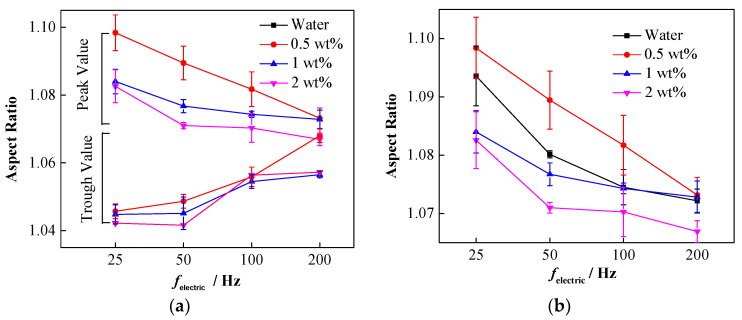
The variation of droplet oscillation amplitude under different particle concentrations and electric field frequencies. (**a**) Oscillation amplitude of nanoparticle-laden droplets; (**b**) oscillation peaks of droplets with different particle concentrations.

Following the study by Szakáll et al. [61], in this study, the 10 *T* method was used to determine the droplet oscillation frequency (Figure 10a). The total time required for the typical 10 oscillations (i.e., 10 *T*) was counted from the time series to calculate the droplet oscillation frequency for each electric field frequency.

In order to ensure the accuracy of the results, the droplet oscillation frequency was measured at least three times, the average value was calculated, and the corresponding result is shown in Figure 9b. Clearly, the oscillation frequency of the nanoparticle-laden droplets is still close to twice the frequency of the electric field [50], and varying the concentration of NPs has little effect on the deformation frequency of the droplet.

It was found that the deformation degree of the nanoparticle-laden droplets decreased with the increase in the frequency. The deformation peaks and trough values in the last oscillation period before the droplet made contact with the interface were considered for further analysis. Figure 10a shows that with the increase in the frequency of the electric field, the decreased peak values and increased trough values continue to approach, which indicates a decrease in the oscillation amplitude. Moreover, the concentration of the NPs also affects the deformation amplitude of the droplets. Figure 10b shows the deformation peaks of nanoparticle-laden droplets at different concentrations. Clearly, under different electric field frequencies, the 0.5 wt% droplets exhibited the largest deformation peak, followed by the 1 wt% droplets, and that of the 2 wt% droplets was the smallest. This indicates that increasing the concentration of NPs suppresses the oscillating deformation of droplets in an AC electric field. Compared to pure droplets, the 0.5 wt% droplets have a larger oscillation deformation peak in the last period before making contact with the interface, which is related to the reduction of the interfacial tension of the droplets caused by the NPs. However, the oscillation deformation peaks of the 1 and 2 wt% droplets become smaller, which is caused by the rigid particle interface film on the droplet surface.

**Figure 11:**
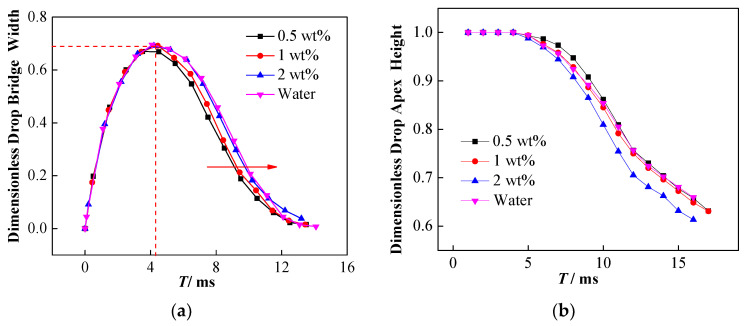
Effect of NPs on droplet bridge topological changes. (**a**) Effect of NP concentration on the development of dimensionless liquid bridge width; (**b**) effect of NP concentration on dimensionless droplet apex height.

The change of interfacial tension affects not only the droplet falling process, but also the affects how the droplet morphology changes during the coalescence process. Figure 11a shows that increasing the concentration of NPs can hinder the shrinkage of the liquid bridge to a certain extent. In other words, the dimensionless width of the liquid bridge for high concentration NP droplets is larger, which is conducive to the drainage of the droplets, thereby inhibiting partial coalescence and secondary droplet formation.

The presence of NPs can also affect the vertical collapse process of the droplets. Figure 11b exhibits the effect of NP concentration on the vertical collapse of coalesced droplets. The results indicate that the 2 wt% nanoparticle-laden droplets show the lowest dimensionless droplet apex height during coalescence, while the 0.5 wt% nanoparticle-laden droplets exhibit the highest one. The dimensionless height at the apex of the 1 wt% nanoparticle-laden droplets is close to that of pure water droplets and is slightly lower than that of 0.5 wt% nanoparticle-laden droplets. The influence of the NPs may be related to the surface activity of the particles. Two types of influence are observed when the NPs are adsorbed onto the oil–water interface: one is the formation of a particle film with a certain strength, and the other involves the reduction in the oil–water interfacial tension. Moreover, a competitive relationship exists between these two effects on the vertical collapse of droplets. The 0.5 wt% nanoparticle-laden droplets show the lowest interfacial tension and are easily stretched and deformed under the force of the electric field, which may be the main cause of the hindering of its vertical collapse. Comparative analysis of 1 wt% nanoparticle-laden and pure water droplets indicates that although the reduced interfacial tension promotes deformation, the interfacial particle film hinders deformation. Consequently, an insignificant effect of the particles is observed on the vertical collapse for the balance of these two effects. In contrast, for the 2 wt% nanoparticle-laden droplets, even if the interfacial tension is close to that of pure water droplets, the formation of the interfacial particle film still promotes its vertical collapse. Thus, the competition of these two effects is the reason for different droplet apex dimensionless height.

### 4.2. Effect of Conductivity

**Figure 12:**
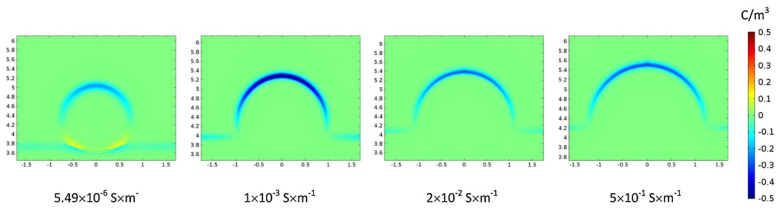
Changes in surface charge density of the droplet for different conductivity [62].

It was found that after the SiO_2_ NPs were adsorbed onto the oil–water interface, the negative charge density at the interface increased. Table 1 summarizes that the introduction of NPs can significantly improve the conductivity of the water phase. Studies have shown that increasing the conductivity of the water phase to a certain value can inhibit the formation of secondary droplets [62]. Figure 12 demonstrates that when the conductivity of the water phase exceeds 1 × 10^−3^ S m^−1^, as the electrical conductivity continues to increase, the space charge density on the upper part of the droplet decreases, which in turn leads to a decrease in the force of the electric field that is applied to the droplet. This decreased electric field force is not conducive to partial coalescence. In this study, with the increase in the concentration of NPs from 0.5 to 2 wt%, the conductivity increased from 3.12 × 10^−3^ to 1.086 × 10^−2^ S m^−1^; thus, an increase in conductivity in this interval inhibits the formation of secondary droplets, which is consistent with the experimental results.

However, for coalescence systems containing NPs, the charge transfer and distribution between the droplet–interface is more complicated. In general, there are two ways, i.e., polarized charge exchange and charged species transport, for the charge transfer in the process of droplet–interface coalescence. The speed of charge exchange is determined by the charge relaxation time *τ*, where the charge relaxation time of the liquid phase is expressed as follows:

(15) $$\tau =\frac{\varepsilon_{\mathrm{water}}}{\kappa_{\mathrm{water}}}$$

where *ε*_water_ and *κ*_water_ represent the permittivity and the conductivity of water phase, respectively.

For the oil–water system between flat electrodes, the system charge relaxation time can be expressed as follows [63]:

(16) $$\tau =\frac{\varepsilon_{\mathrm{oil}}H_{\mathrm{oil}}{+\varepsilon }_{\mathrm{water}}H_{\mathrm{water}}}{\kappa_{\mathrm{oil}}H_{\mathrm{water}}+\kappa_{\mathrm{water}}H_{\mathrm{oil}}}$$

where *κ*_oil_ represents the conductivity of oil phase.

Therefore, the introduction of NPs leads to an increase in the conductivity of the water phase, resulting in a shorter charge relaxation time, which indicates the acceleration in the polarization charge transfer speed in the coalescence system. Moreover, as a charged medium, the NPs also participate in charge transfer when they move with the fluid in the coalescence process. For instance, Hamlin et al. [64] confirmed that the convection of liquids containing ions plays an important role in the charge transfer process. However, compared with ions such as Na^+^ and Cl^−^, NPs are larger in mass and volume, and thus consume more kinetic energy in the process of coalescence into the bulk liquid, and the migration speed is slower as well. Moreover, owing to the long falling time of the droplets, the charged NPs have sufficient time to redistribute under an external electric field, resulting in the fact that the actual state of charge on the nanoparticle-laden droplets during coalescence is different from the above-mentioned theoretical prediction, which may weaken the influence of conductivity.

**Figure 13:**
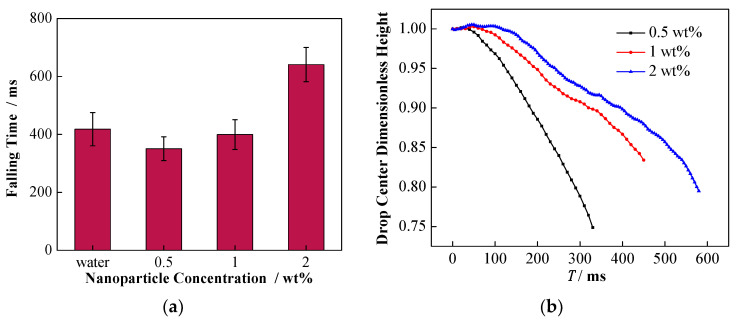
Effect of NP concentration on the migration of falling droplet. (**a**) The time of the droplet center falling to the interface under different particle concentrations; (**b**) effect of NP concentration on the dimensionless droplet center height.

In this case, the effect of the change of conductivity by adding NPs can be reflected in the falling process as well as the formation of secondary droplets. Specifically, the influence of NPs on the migration of falling droplet is shown in Figure 13. For the three different concentrations of nanoparticle-laden droplets, as shown in Figure 13a, the falling time of the 2 wt% nanoparticle-laden droplets was the longest. Moreover, Figure 13b illustrates that the dimensionless height of the center for the 0.5 wt% nanoparticle-laden droplets decreased the most rapidly, followed by that the of 1 wt%, and the decrease of the 2 wt% was the slowest. The migration of nanoparticle-laden droplets should be related to their complex forces. As mentioned above, the falling liquid droplet is driven to the interface by gravity and the electrostatic attraction between the droplet and the interface. However, the nanoparticle-laden droplets are negatively charged, and the droplets are exposed to the upward electric field force provided by the positive plate, which can hinder the falling of the droplets. With the increase in the particle concentration, the charge capacity of the droplets also increases, which results in greater resistance to falling, caused by the electric field.

**Figure 14:**
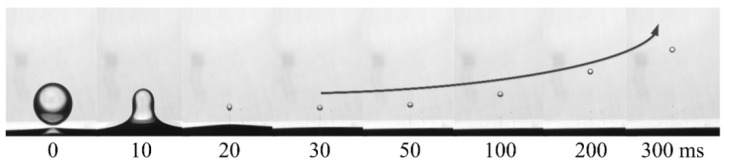
Rising process of secondary droplet in a DC electric field.

Owing to the redistribution of charge, charge aggregation may occur on the upper part of the droplet, and the actual charge on the upper part of the coalescing droplet is eventually reflected by the formed secondary droplets. Figure 14 exhibits that the secondary droplet has a net charge and moves up to the high potential area under the action of the electric field.

**Figure 15:**
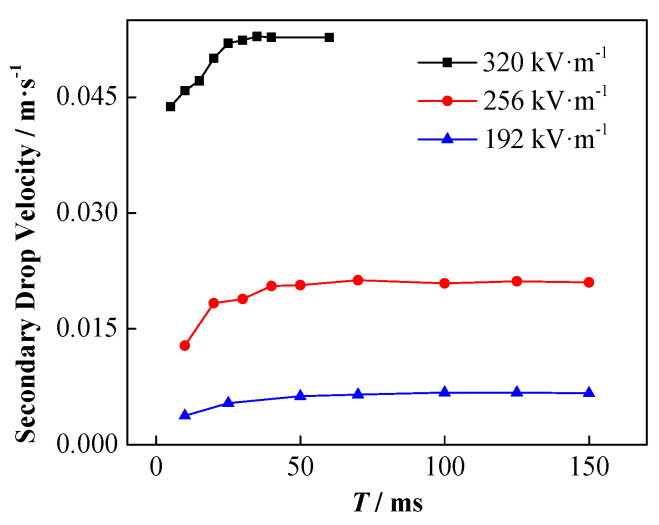
Change in the rising speed of secondary droplets with time under different electric field strength conditions (the particle concentration is 1 wt%).

In the DC electric field, the change of the increasing speed of the secondary droplets and the influence of the applied electric field strength are shown in Figure 15. Among them, the initial droplet diameter is 1241 µm, and the initial height is 0.5 mm. Clearly, the secondary droplets are driven by the force of the electric field to accelerate rising at the beginning. With the increase in the moving speed of the secondary droplets, the viscous resistance it receives also increases, and the overall force on the droplet gradually reaches equilibrium. Thereafter, the secondary droplets continue to move at a certain stable speed. The acceleration of the secondary droplets increases with the enhancement of the applied electric field thus, they reach a greater stable speed sooner.

**Figure 16:**
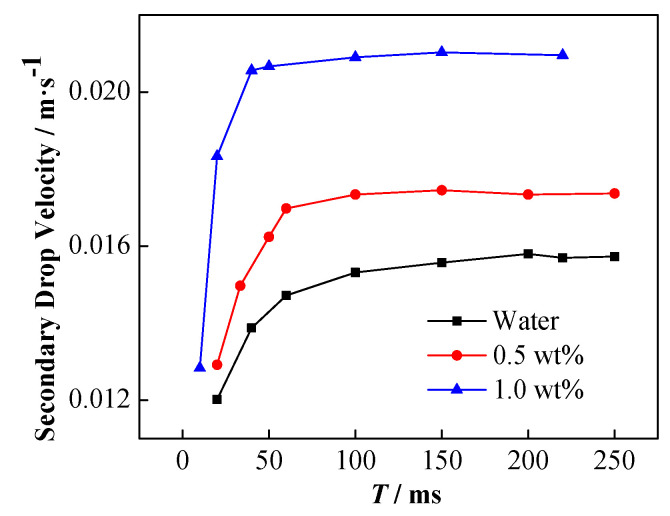
Change of the rising speed of secondary droplets with time under different NP concentration conditions, electric field strength is 256 kV·m^−1^.

The influence of NP concentration on the velocity of the secondary droplets is shown in Figure 16. The higher the particle concentration is, the faster the acceleration of the secondary droplets is, and the greater the stable rising speed is.

The increase in the charge of the secondary droplet results in the increase of the rising speed. The charge capacity of the secondary droplets can be calculated by using the force balance. The secondary droplets with a stable rising speed are subject to the combined action of gravity *G*, buoyancy *F*_b_, drag force *F*_d_, and electric field force *F*_E_ thus, the force balance equation is expressed as follows:

*F*_E_ + *F*_b_ = *F*_d_ + *G* (17)

Considering the expressions of each force, it can be modified as

(18) $$EQ=\frac{1}{2}\rho_{c}C_{d}Av^{2}{+(\rho }_{c}{-\rho }_{d})Vg$$

**Figure 17:**
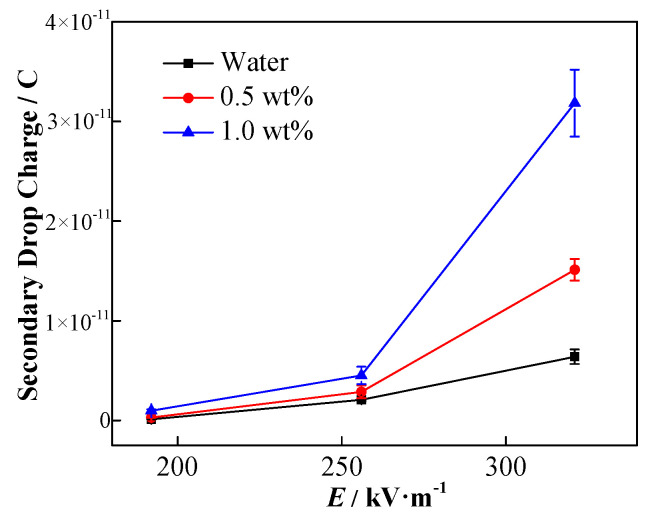
Effects of electric field strength and NP concentration on secondary droplet charge.

Figure 17 shows the final secondary droplet charge calculated according to Equation (18).

The charge of the secondary droplets increases with the enhancement of the applied electric field. Therefore, it can be inferred that the greater the amount of charge on the upper part of the coalescing droplets, the stronger the force of the electric field that is applied to the droplets during coalescence. Owing to the electronegative nature of NPs, the elevated NP concentration results in the increment of secondary droplets charge. The result depicts that not only the initial droplet contains negative NPs, but it may also verify the previous perspective that under the external electric field, more particles coalesce on top of the coalesced droplets and eventually remain in the secondary droplets. Therefore, the above-mentioned results indicate that the increase in liquid phase conductivity may not be the main reason for the inhibition of the formation of secondary droplets as predicted by numerical simulation in the case of the stationary droplet–interface coalescence. Owing to the NP distribution, the charge accumulates on the upper part of the droplet, which facilitates the formation of secondary droplets. As a result, the reduction of secondary droplet formation with the incremental addition of the NP concentration may mainly be determined by the interfacial tension change.

## 5. Conclusions

In this study, the effects of different concentrations of NPs on droplet–interface coalescence were studied using a high-speed camera. It was found that the interfacial tension first decreased and then increased, while the conductivity increased with the increase in the NP concentration. In the experiment, the droplet experienced a fall, coalescence and secondary droplet formation.

It was found that the secondary droplet formation was inhibited with increasing NP concentration. This result is attributed to the interfacial tension and conductivity change. For the droplet deformation while falling, the decrease of interfacial tension facilitates it; however, the interfacial film formed in high particle concentrations can inhibit it. In alternating current electric field, particle concentration has little effect on droplet oscillation frequency, and droplet deformation is inhibited with the increase of electric field frequency or particle concentration. Moreover, particle concentration also leads to the changes in the shape of the liquid bridge. With the increase of particle concentration, the horizontal contraction of the liquid bridge was inhibited thus, it did not facilitate the formation of secondary droplets. Owing to the competition between the interfacial film and the interfacial tension, the dimensionless droplet apex height decreased with the increase of the concentration, which promoted the vertical collapse of the droplet.

Owing to conductivity change with the NPs, the actual charge transfer and distribution between the droplet–interface interaction is complicated. The velocity of the falling droplet decreases, while the rising velocity of the secondary droplet increases with the increase of particle concentration under an electric field. The inverse calculation of the force balance equation shows that the charge of the secondary droplet increases with increased NP concentration. On the one hand, this is due to the high concentration of negative NPs in the initial droplet. On the other hand, it may also verify that charged NPs have sufficient time to redistribute, resulting in charge accumulation on the top of the droplet. The conductivity change does not significantly inhibit secondary droplet formation due to the NP thus, the interfacial tension change is the main factor.

Overall, the research involves the experimental study of the influence of NPs on droplet–interface electro-coalescence. We have initially explored the effects of interfacial tension and conductivity with different NP concentrations on droplet deformation, migration, coalescence, and secondary droplets. However, there are still many issues that have not yet been clarified, such as the underlying mechanism of NP movement under the electric field and the charge distribution during coalescence. As a result, a numerical simulation can be used to further study the topological changes to the droplet bridge and charge distribution during droplet–-interface coalescence.

## Figures and Tables

**Table 1:** Physical properties of deionized water and silicon oil.

	Conductivity (μS·cm^−1^)	Viscosity (mPa·s)	Density (kg·m^−3^)	Interfacial Tension (mN·m^−1^)
Deionized water	1.57	1.046	1000	35.68
Dimethyl silicone oil	2.3 × 10^−7^	156.1	963	
0.5 wt%	31.2	1.089	1015	4.56
1 wt%	58.3	1.124	1016	9.57
2 wt%	108.6	1.212	1020	34.14

## Data Availability

The data presented in this study are available on request from the corresponding author.

## Supplementary Materials

The following are available online at https://www.mdpi.com/article/10.3390/nano11071764/s1, Video S1: 0.5 wt%.
